# HGFAC is a ChREBP-regulated hepatokine that enhances glucose and lipid homeostasis

**DOI:** 10.1172/jci.insight.153740

**Published:** 2023-01-10

**Authors:** Ashot Sargsyan, Ludivine Doridot, Sarah A. Hannou, Wenxin Tong, Harini Srinivasan, Rachael Ivison, Ruby Monn, Henry H. Kou, Jonathan M. Haldeman, Michelle Arlotto, Phillip J. White, Paul A. Grimsrud, Inna Astapova, Linus T. Tsai, Mark A. Herman

**Affiliations:** 1 Duke Molecular Physiology Institute, Duke University, Durham, North Carolina, USA.; 2 Division of Endocrinology, Diabetes and Metabolism, Beth Israel Deaconess Medical Center, Harvard University, Boston, Massachusetts, USA.; 3 Harvard Medical School, Boston, Massachusetts, USA.; 4 Division of Endocrinology, Metabolism, and Nutrition, Department of Medicine, and; 5 Department of Pharmacology and Cancer Biology, Duke University, Durham, North Carolina, USA.

**Keywords:** Metabolism, Carbohydrate metabolism, Glucose metabolism, Growth factors

## Abstract

Carbohydrate response element–binding protein (ChREBP) is a carbohydrate-sensing transcription factor that regulates both adaptive and maladaptive genomic responses in coordination of systemic fuel homeostasis. Genetic variants in the ChREBP locus associate with diverse metabolic traits in humans, including circulating lipids. To identify novel ChREBP-regulated hepatokines that contribute to its systemic metabolic effects, we integrated ChREBP ChIP-Seq analysis in mouse liver with human genetic and genomic data for lipid traits and identified hepatocyte growth factor activator (HGFAC) as a promising ChREBP-regulated candidate in mice and humans. HGFAC is a protease that activates the pleiotropic hormone hepatocyte growth factor. We demonstrate that HGFAC-KO mice had phenotypes concordant with putative loss-of-function variants in human HGFAC. Moreover, in gain- and loss-of-function genetic mouse models, we demonstrate that HGFAC enhanced lipid and glucose homeostasis, which may be mediated in part through actions to activate hepatic PPARγ activity. Together, our studies show that ChREBP mediated an adaptive response to overnutrition via activation of HGFAC in the liver to preserve glucose and lipid homeostasis.

## Graphical abstract

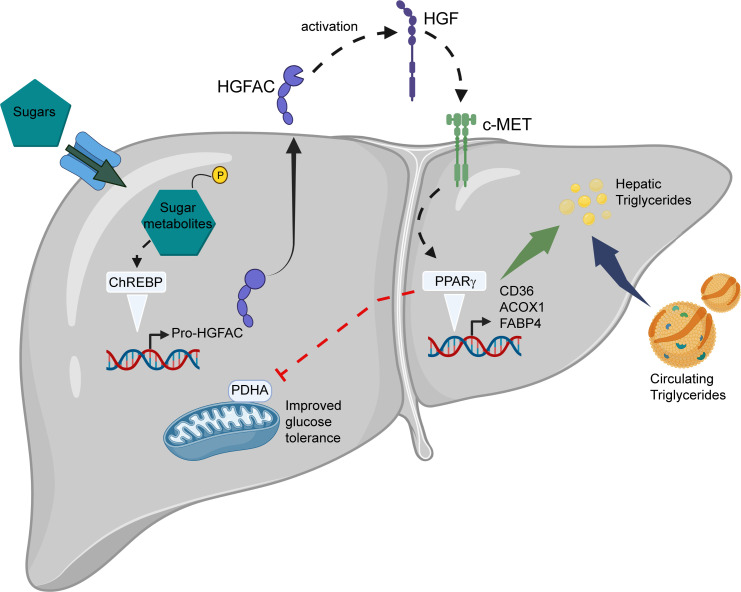

## Introduction

Carbohydrate response element–binding protein (ChREBP, also known as MLXIPL) is a transcription factor expressed in key metabolic tissues including liver, adipose tissue, kidney, small intestine, and pancreatic islets (1, 2). It is activated by sugar metabolites, and in the liver and small intestine, it is robustly activated following fructose ingestion (3, 4). Activated ChREBP stimulates expression of genomic programs contributing to adaptive and maladaptive metabolic responses (1). Hepatic ChREBP activity is increased in human obesity and diabetes (5, 6). Knockdown or knockout of hepatic ChREBP protects against metabolic disease in diet and genetic forms of obesity (3, 7, 8).

ChREBP plays a significant role in human metabolic physiology. Common genetic variants in the *ChREBP* locus associate with pleiotropic metabolic traits including circulating lipids and cholesterol, BMI, waist-hip ratio, height, diverse hematological parameters, serum urate, liver enzymes, and blood pressure (9). The complement of ChREBP transcriptional targets that regulates these diverse traits is incompletely understood. To date, ChIP-Seq assays have implicated thousands of genes as ChREBP targets (10, 11). It is well established that ChREBP regulates glycolysis and fructolysis, hepatic and adipose lipogenesis, and hepatic glucose production via regulation of key enzymes involved in these metabolic pathways (4, 12–14). At the same time, most putative ChREBP transcriptional targets have unknown or poorly defined metabolic impact.

We performed ChIP-Seq for ChREBP in mouse liver and integrated this with human genetic data to identify ChREBP-dependent hepatokines that might regulate systemic metabolism. Through this screen we identified hepatocyte growth factor activator (HGFAC) as a promising candidate. HGFAC is a liver-secreted, circulating protease that activates hepatocyte growth factor (HGF), which regulates pleiotropic biological activities, including morphogenesis, cell migration, cell state transition, and proliferation in epithelial and other cell types throughout the body (15–17). We demonstrate that HGFAC is indeed nutritionally regulated in a ChREBP-dependent manner and participates in an adaptive response maintaining carbohydrate and lipid homeostasis.

## Results

### HGFAC is a ChREBP genomic target associating with metabolic traits in humans.

**Figure 1:**
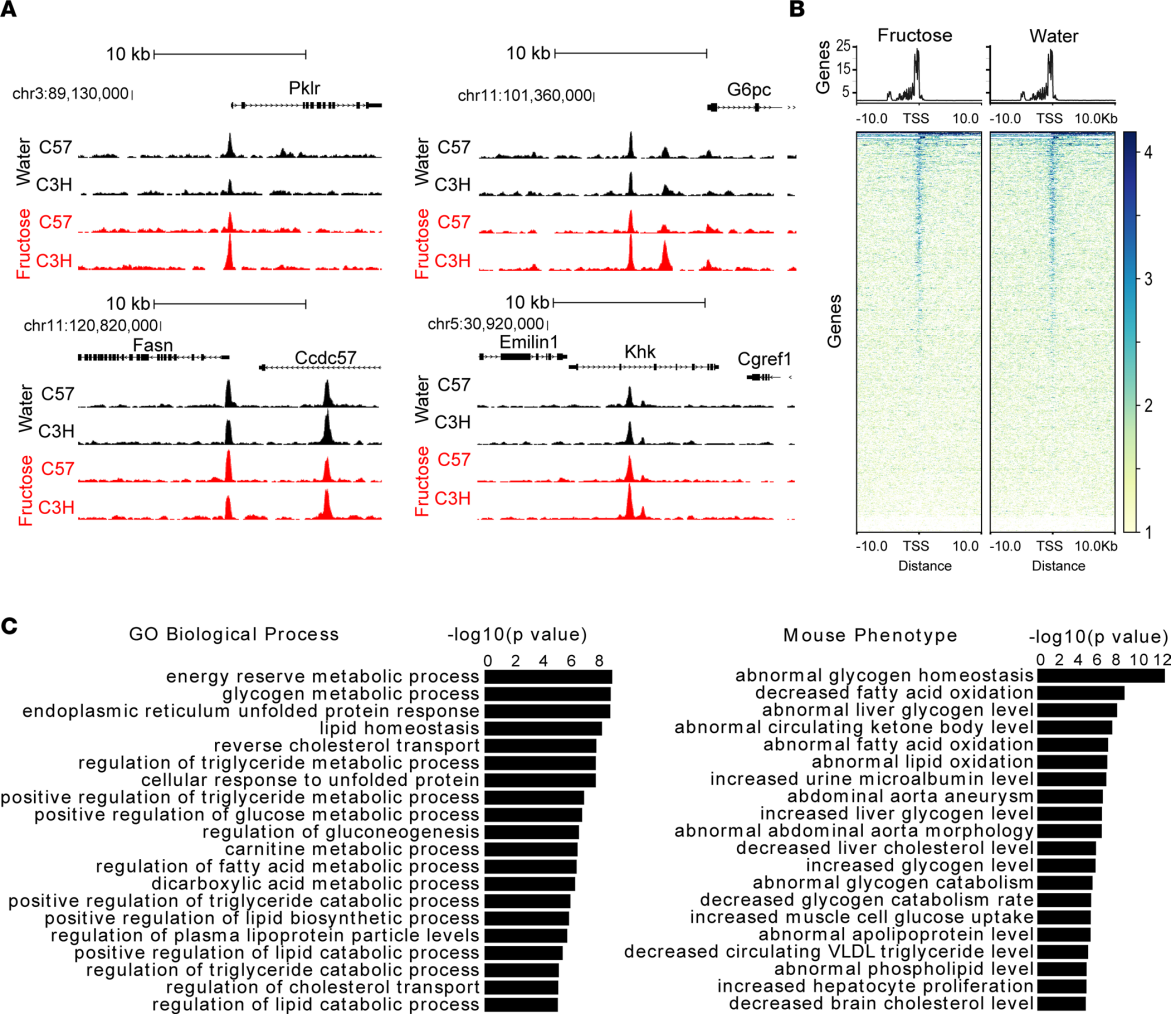
ChREBP is bound to liver genomic targets following water and fructose gavage after a 5-hour fast. (**A**) ChREBP ChIP-Seq signal tracks in liver of male C57 and C3H mice after a 5-hour fast and 90 minutes after water versus fructose gavage (4 g/kg body weight) in selected ChREBP transcriptional targets including Pklr, Gp6c, Fasn, and Khk. (**B**) Heatmaps showing hepatic ChREBP peaks after fructose versus water gavage. The amplitude of each peak center is represented by the *z* score and shown in blue. TSS, transcriptional start site. (**C**) Gene Ontology (GO) biological process and mouse phenotype pathway analysis for ChREBP peaks.

To identify ChREBP transcriptional targets that participate in the regulation of ChREBP-associated metabolic programs and phenotypes, we performed ChIP-Seq analysis for ChREBP in livers of 2 strains of male mice gavaged with either water or fructose. We identified 4,860 distinct genomic sites enriched for ChREBP binding (Supplemental Table 1; supplemental material available online with this article; https://doi.org/10.1172/jci.insight.153740DS1), which include well-defined loci in canonical ChREBP targets involved in glycolysis, glucose production, fructolysis, and lipogenesis, such as liver pyruvate kinase (PKLR), glucose-6-phosphatase (G6PC), fatty acid synthase (FASN), and ketohexokinase (KHK), respectively (Figure 1A). Although fructose gavage can acutely induce ChREBP-dependent changes in gene expression, ChREBP ChIP-Seq peaks were readily detectable in fasted mice, and fructose gavage did not enhance ChREBP ChIP-Seq peak height even at a liberal FDR of 0.20. This indicates that increased chromatin occupancy is not essential for fructose to induce ChREBP-dependent gene transcription. Most ChREBP ChIP peaks occurred within 10 kb of transcriptional start sites (Figure 1B). Consistent with ChREBP’s known functions, Genomic Region Enrichment Analysis of putative ChREBP binding sites demonstrated enrichment for numerous metabolic processes, including carbohydrate and lipid metabolism (Figure 1C) (18).

Variants in the *ChREBP* locus are strongly associated with hypertriglyceridemia in human populations (19, 20). However, the complement of ChREBP transcriptional targets that mediate its effects on circulating lipids is uncertain. We sought to determine whether genomic loci containing human homologs of mouse ChREBP target genes are enriched for variants that associate with hypertriglyceridemia in human populations. Via Meta-Analysis of Gene-set ENrichmenT of variant Associations (MAGENTA), we demonstrated that loci in proximity to human homologs of mouse genes that were within 20 kb of ChREBP binding sites were enriched for SNPs that associate with hypertriglyceridemia in humans (adjusted *P* = 0.003). A total of 87 loci/genes contributed to this enrichment with an FDR of 0.05 (Table 1 and Supplemental Table 2) (21). This list includes known ChREBP transcriptional targets, such as *GCKR*, *TM6SF2*, *KHK*, and *ChREBP* (*MLXIPL*) itself, all previously implicated in regulating carbohydrate and triglyceride metabolism. Of these 87 loci, 7 encoded putative secretory proteins, including several lipoproteins (APOC2, APOE, and APOA5); VEGFA, which is most well known for its role in angiogenesis but also implicated in metabolic control; and HGFAC (Supplemental Table 2) (22). To our knowledge, HGFAC has neither been identified as a ChREBP transcriptional target nor been studied extensively in the context of systemic fuel metabolism.

HGFAC is a serine protease expressed predominately in hepatocytes and secreted as a zymogen into circulation, where it is found in a single-chain pro-HGFAC form (23, 24). In vitro studies have identified thrombin and kallikrein-related peptidases KLK-4 and KLK-5 to be potent activators of pro-HGFAC (25, 26). Once activated, HGFAC cleaves and activates HGF, which can then bind and activate the c-Met receptor tyrosine kinase (c-MET) (23). HGF and c-MET have pleiotropic biological activities as mitogens and motogens in organogenesis, tissue repair, and cell migration, and function as antiinflammatory, apoptotic, and cytoprotective signals depending on the context (15). Variants in *c-MET* also associate with circulating triglycerides at genome-wide significance in humans, consistent with a potential role for HGFAC in regulating triglyceride levels through activation of HGF (27). Moreover, increased levels of circulating HGF in people associate with features of cardiometabolic disease, including obesity, risk for type 2 diabetes, and risk for cardiovascular disease (28–31). Circulating HGF levels are influenced by variants in the HGFAC locus (32). A missense variant in *HGFAC*, rs3748034, that associates with increased circulating HGF also associates with increased circulating triglycerides in GWAS aggregate data at genome-wide significance (β = 0.0302, *P* < 5 × 10^–28^) as well as other cardiometabolic risk factors and pleiotropic biological traits (33, 34). The Ala218Ser mutation encoded by rs3748034 is predicated to be “possibly damaging” by PolyPhen-2 (35). Furthermore, another missense variant in *HGFAC*, rs16844401, that associates with increased circulating triglycerides also associates with increased coronary artery disease risk (36). These associations motivated further investigation to determine whether ChREBP regulates HGFAC expression and whether this interacts with nutritional status to regulate systemic fuel metabolism and cardiometabolic risk factors.

### Nutritional regulation of HGFAC is ChREBP dependent.

**Figure 2:**
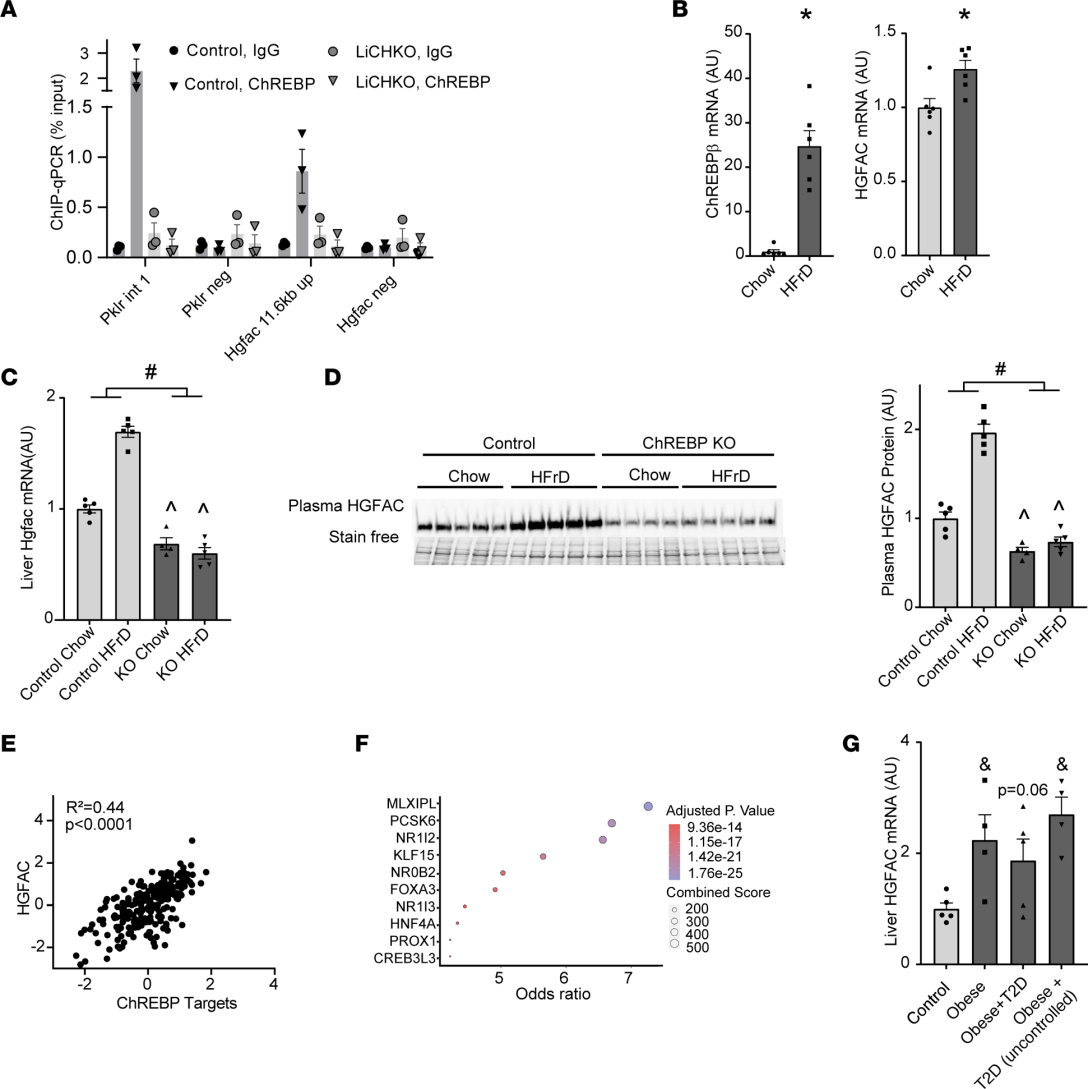
ChREBP links nutritional status to circulating HGFAC. (**A**) ChIP was performed from livers of control and ChREBP-LKO mice with anti-ChREBP or control IgG. qPCR was performed on immunoprecipitated chromatin with primers spanning the E-box in the Pklr promoter and the putative ChREBP binding site in proximity to HGFAC and in nonspecific regions (neg) in proximity to both ChREBP response elements (*n* = 3/group). (**B**) Hepatic *Chrebpβ* and *Hgfac* mRNA expression of overnight-fasted and 4-hour chow- or HFrD-fed Wistar rats (*n* = 7/group). (**C**) Liver mRNA expression and (**D**) circulating levels of HGFAC in control and ChREBP-LKO mice after 8 weeks on chow versus HFrD with densitometric quantification (*n* = 4–5/group). (**E**) Correlation between *HGFAC* mRNA expression and a composite vector comprising canonical ChREBP targets in human livers from the GTEx project (Pearson’s correlation *R*^2^ = 0.44, *P* < 0.0001, *n* = 226). (**F**) Factors ranked by odds ratio for enrichment of the 300 genes most highly coexpressed with the factor in the ARCHS4 project that are also present in the top 5% of genes that correlate with HGFAC expression in the GTEx project. Combined score = log(*P*) × *z*, where *P* is calculated by Fisher’s exact test and *z* score is calculated by assessing the deviation from the expected rank. The size and color of the circles correspond to the enrichment score and adjusted *P* value, respectively. (**G**) Expression of *HGFAC* mRNA in livers of healthy controls, obese nondiabetic participants, and obese participantswith well-controlled diabetes and poorly controlled diabetes, (*n* = 4–5/group). Data represent means ± SEM. Statistics were assessed by 2-way ANOVA with Holm-Šídák multiple comparisons between individual groups, ^#^*P* < 0.05, for main effects, ^*P* < 0.05 for comparison across genotypes within diets; or 1-way ANOVA with Holm-Šídák multiple comparisons test between control and other groups, ^&^*P* < 0.05.

To confirm specific binding of ChREBP to the putative biding site in proximity to the *Hgfac* gene, we performed targeted ChIP–quantitative PCR (ChIP-qPCR) on livers from control and liver-specific ChREBP-KO (ChREBP-LKO) mice with anti-ChREBP and control IgG. The putative binding site was enriched when ChREBP IP was performed on control but not ChREBP-LKO liver samples (Figure 2A). ChREBP activity in the liver is responsive to diets high in fructose. To test whether acute fructose feeding induces hepatic expression of *Hgfac*, we fed overnight-fasted Wistar rats with high-fructose diet (HFrD) or control chow diet for 4 hours and measured hepatic *Chrebpβ* and *Hgfac* mRNA levels. Acute fructose feeding induced *Chrebpβ* expression by more than 20-fold (*P* < 0.0001) while *Hgfac* mRNA levels increased by 25% (*P* < 0.05) (Figure 2B). To examine the role of hepatic ChREBP in the regulation of HGFAC in rodents, we measured hepatic *Hgfac* mRNA and HGFAC protein in the liver and plasma of mice with liver-specific deletion of ChREBP after 8 weeks on HFrD or control diet. High-fructose feeding increased hepatic *Hgfac* mRNA expression 1.7-fold (*P* <.0001) in control mice, and this induction was abrogated in ChREBP-LKO mice (Figure 2C). Fructose-induced increases in hepatic *Hgfac* mRNA expression were accompanied by 4- and 2-fold increases in hepatic and circulating pro-HGFAC protein levels (Figure 2D and Supplemental Figure 1A). Basal liver and circulating HGFAC protein levels tended to be decreased in chow-fed ChREBP-LKO mice and were not induced with fructose feeding. Circulating HGFAC also increased in mice fed a high-fat/high-sucrose (HF/HS) diet and in genetically obese Zucker fatty rats on chow diet (Supplemental Figure 1, B and C), where hepatic ChREBP activity is also robustly increased independently of an obesogenic diet (37). These data show that hepatic ChREBP mediates diet- and obesity-induced increases in circulating HGFAC.

Next, we examined whether the genomic region containing the ChREBP binding site in proximity to *Hgfac* in mice (chr5: 35,029,873–35,030,157) is conserved in humans. Analysis using the UCSC Genome Browser demonstrated that the corresponding region in the human genome was highly homologous to the mouse region, with 76.8% of nucleotide identity, while in rats this homology achieved 99.6% identity (38). Next, we sought to determine whether ChREBP-mediated regulation of HGFAC might be conserved in humans. To that end, we analyzed hepatic mRNA expression levels of *HGFAC* and other ChREBP transcriptional targets in the Genotype-Tissue Expression (GTEx) project (39). Expression of the potent *ChREBP-β* isoform is an excellent surrogate marker of tissue ChREBP activity (14). However, it is expressed at low levels, which are typically below the sequencing depth of most RNA-Seq experiments. Consistent with this, GTEx RNA-Seq data do not distinguish between *ChREBP*-*β* and -*α* isoforms. Due to the lack of *ChREBP-β*–specific expression data, we used a composite expression vector comprising 5 well-validated ChREBP target genes (*FASN*, *PKLR*, *KHK*, *ALDOB*, and *SLC2A2*) and found that this composite vector strongly correlated with the expression of *HGFAC* (Pearson’s correlation *R*^2^ = 0.44, *P* < 0.0001) (Figure 2E). Transcription factor enrichment analysis of the 5% of hepatic genes that best correlated with hepatic *HGFAC* expression in the GTEx project showed strong enrichment for genes coexpressed with ChREBP (adjusted *P* = 1.75 × 10^–25^), indicating conservation of the ChREBP-mediated regulation of HGFAC in humans (Figure 2F) (40). Additionally, hepatic *HGFAC* mRNA expression is upregulated in patients with obesity and uncontrolled diabetes (Figure 2G), conditions that are associated with increased hepatic ChREBP activity (5, 41). Collectively, these data support the hypothesis that hepatic HGFAC expression and circulating levels of HGFAC are regulated by ChREBP activity both in rodents and in humans, and hepatic *HGFAC* expression is increased in obesity and diabetes.

### Murine HGFAC KO recapitulates the phenotype of putative human loss-of-function HGFAC variants.

**Figure 3:**
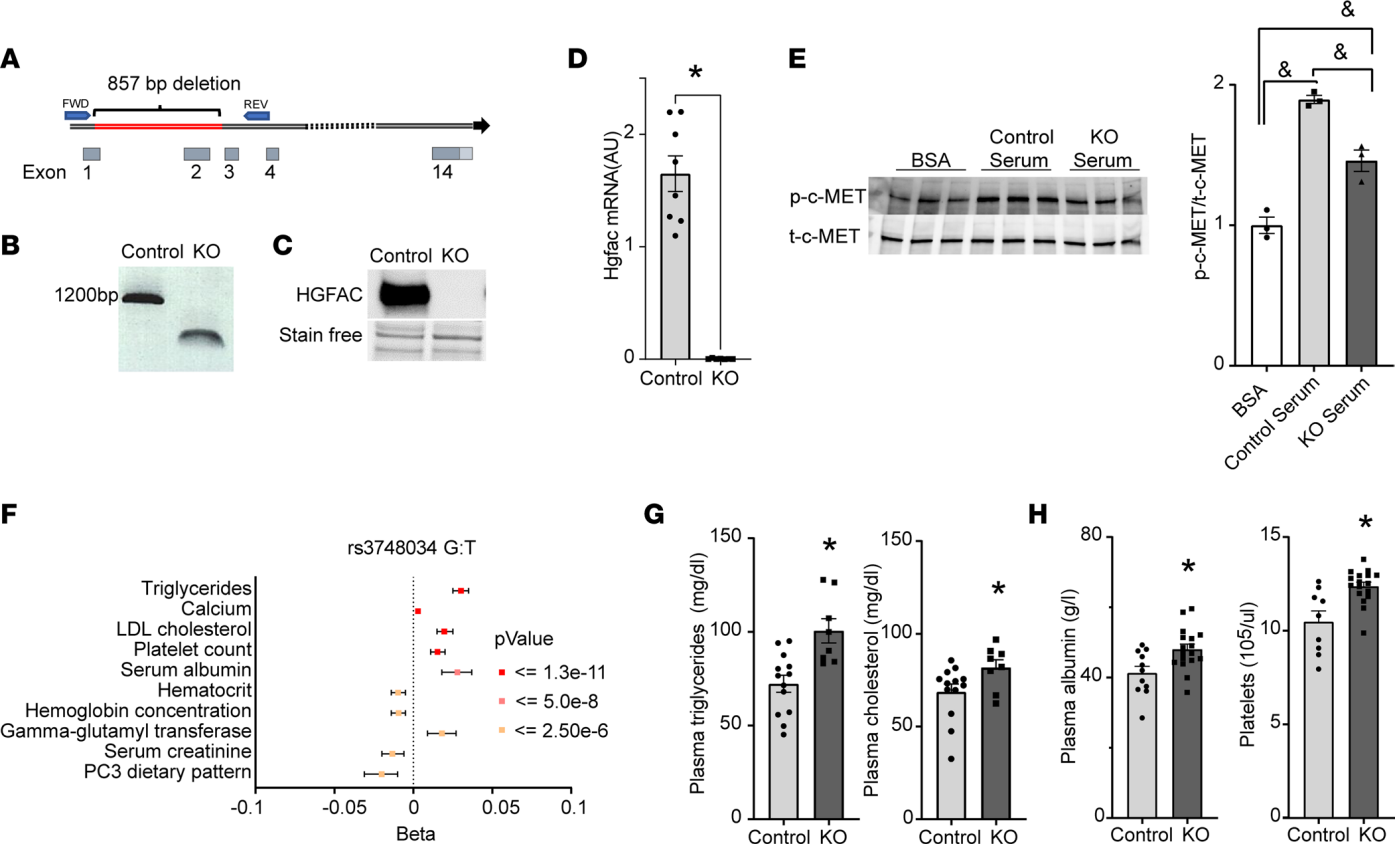
The phenotype in HGFAC-KO mice recapitulates the phenotype of a putative loss-of-function variant in human HGFAC. (**A**) Schematic depiction of *Hgfac* gene and the deleted region in red; FWD and REV indicate the positions of forward and reverse primers, respectively, used in genomic PCR shown in (**B**) confirming the deletion of an 857 bp region in the *Hgfac* gene. (**C**) Representative immunoblot of circulating HGFAC in control (wild-type, littermate control) and KO (HGFAC KO) plasma. (**D**) Hepatic *Hgfac* mRNA levels measured by qPCR in control and HGFAC-KO mice (*n* = 7–9/group). (**E**) Immunoblot and quantification of phosphorylated c-MET in HepG2 cells treated with activated sera of control and HGFAC-KO mice (*n* = 3/condition). (**F**) Forest plot of phenotypes associated with the rs3748034 putative loss-of-function coding variant in human *HGFAC*. (**G**) Quantification of plasma triglyceride and cholesterol levels in ad libitum chow-fed male control and HGFAC-KO mice (*n* = 8–13/group), (**H**) plasma albumin concentrations in male control and HGFAC-KO mice (*n* = 11–17/group), and plasma platelet levels in male control and HGFAC-KO mice (*n* = 9–17/group). Data represent means ± SEM. Statistics were assessed by 2-tailed unpaired *t* test, *P < 0.05; or 1-way ANOVA with Holm-Šídák multiple comparisons test between groups, ^&^*P* < 0.05.

To study the roles of HGFAC in systemic metabolism, we generated whole-body HGFAC-KO mice that lacked a portion of exon 1 and all of exon 2 (Figure 3A). The deletion was confirmed by genomic PCR, by the absence of detectable circulating HGFAC protein, and by quantification of hepatic *Hgfac* mRNA (Figure 3, B–D). HGFAC-KO mice were born at normal Mendelian ratios and did not appear to have any gross abnormalities when compared with their littermate controls. Activated HGFAC activates HGF and c-MET signaling. However, there is redundancy in this system, and other proteases including hepsin (HPN) and coagulation factors XIa and XIIa are also capable of activating HGF (42, 43). We sought to determine whether the ability to activate endogenous HGF is impaired in HGFAC-KO mice. c-MET signaling was assessed in HepG2 cells incubated with thrombin-treated sera obtained from control and HGFAC-KO mice. Thrombin is one of the proteases that is capable of activating HGFAC in vitro (26). Serum from control mice increased c-MET phosphorylation 1.9-fold when compared with controls (DMEM+BSA), while this induction was attenuated with serum from KO mice (Figure 3E). These results demonstrate that sera from HGFAC-KO mice have reduced capacity to activate HGF and c-MET signaling.

A putative loss-of-function variant in *HGFAC* (rs3748034) strongly associated with increased circulating triglycerides, LDL-cholesterol, albumin, and platelets, among other traits (Figure 3F) (33). We determined whether HGFAC-KO mice had similar phenotypes. Male HGFAC-KO mice had a 28% increase in circulating triglycerides (mean ± SEM 100 ± 6.5 mg/dL vs. 72 ± 4.5 mg/dL, *P* < 0.001) and cholesterol (82 ± 11.5 mg/dL vs. 69 ± 14.8 mg/dL, *P* < 0.05) (Figure 3G). Similarly, high levels of circulating triglycerides were present in female HGFAC-KO mice (89 ± 5.4 mg/dL vs. 65 ± 3 mg/dL, *P* < 0.005) (Supplemental Figure 2A). These differences were detected in the ad libitum–fed but not in overnight-fasted condition, while nonesterified fatty acids were similar between the groups in both ad libitum–fed and fasted states (Supplemental Figure 2, B and C). Additionally, HGFAC-KO mice had a 15% increase in circulating albumin (4.8 ± 0.19 g/dL vs. 4.1 ± 0.15 g/dL, *P* < 0.01) and a 15% increase in circulating platelets (1,237 ± 22 cells × 10^3^/μL vs. 1,048 ± 57 cells × 10^3^/μL, *P* < 0.05) (Figure 3H). No hematological parameter other than platelet count was altered (Supplemental Figure 2D). Collectively, these data indicate that murine HGFAC KO recapitulates phenotypes in putative loss-of-function human *HGFAC* variants.

### HGFAC-KO mice develop impaired glucose homeostasis.

**Figure 4:**
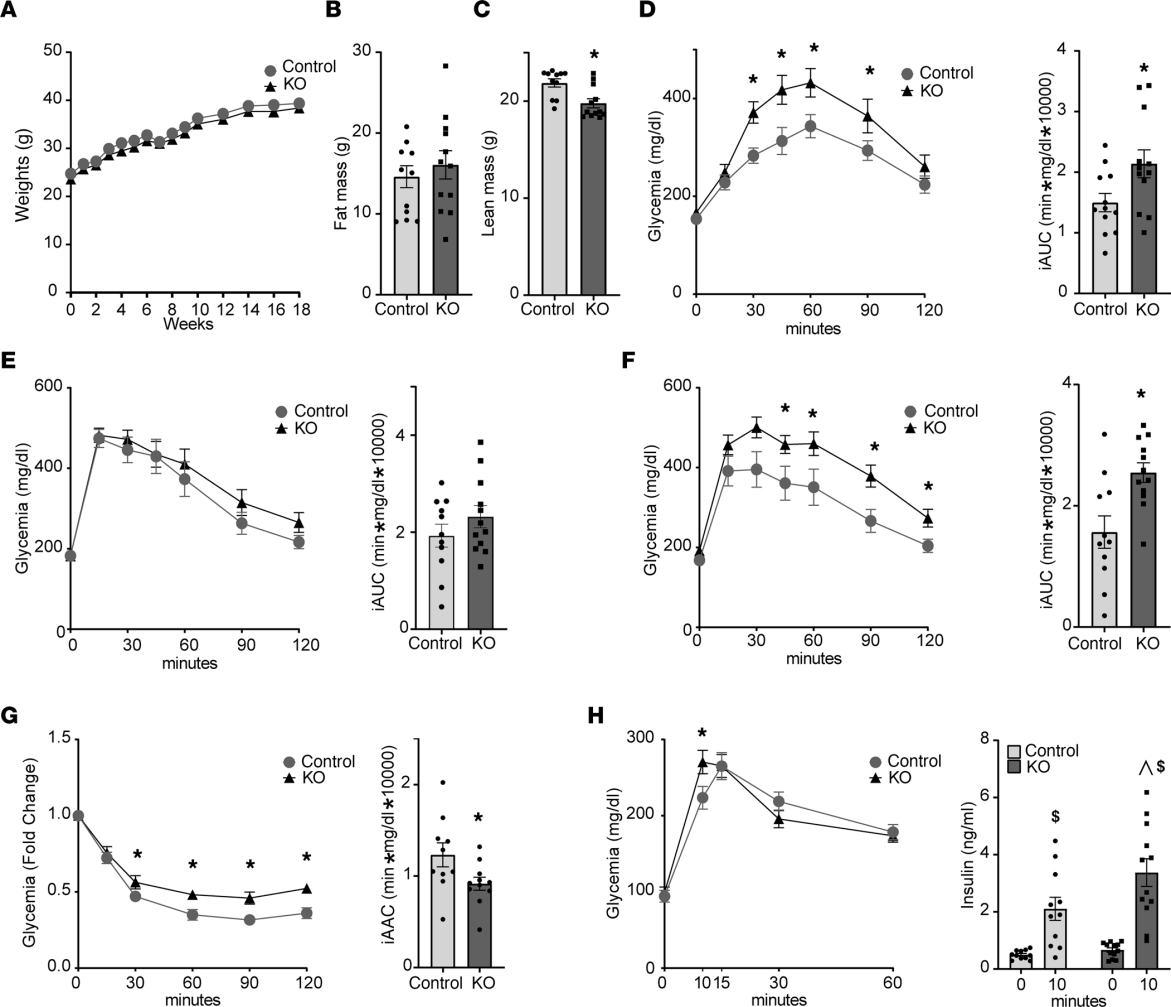
HGFAC-KO mice have impaired carbohydrate metabolism on HF/HS diet. (**A**) Body weight of male control and HGFAC-KO mice during 18 weeks of HF/HS feeding (*n* = 11–12/group unless otherwise specified). (**B**) Fat and (**C**) lean mass by NMR at 18 weeks. Glucose homeostasis was assessed at intervals throughout the study including (**D**) IP glycerol tolerance test at 4 weeks (**E**) IP glucose tolerance at 5 weeks, (**F**) IP glucose tolerance test at 13 weeks, (**G**) IP insulin tolerance test at 14 weeks (*n* = 10–11/group), and (**H**) a mixed meal tolerance test to assess insulin secretion at 16 weeks. Tail vein insulin levels were measured at 0 and 10 minutes. Data represent means ± SEM. Statistics were assessed by 2-tailed unpaired *t* test, *P < 0.05; or 2-way ANOVA with Holm-Šídák multiple comparisons between individual groups, ^*P* < 0.05 for comparison across genotypes within time points, ^$^*P* < 0.05 for comparison across time points within genotypes.

To examine the potential role of HGFAC in systemic metabolism, we challenged 8-week-old HGFAC-KO mice and their littermate controls with HF/HS diet for 18 weeks. We did not observe any differences in body weight or fat mass during the study (Figure 4, A and B). However, a modest reduction in lean body mass was observed in HGFAC-KO mice (Figure 4C). To assess glucose homeostasis, we performed glucose and glycerol tolerance tests in HGFAC-KO mice and controls at time points throughout the study. Glycerol is a preferred gluconeogenic substrate, and glycerol tolerance tests reflect hepatic glucose production capacity (44). After 4 weeks on HF/HS diet, HGFAC-KO mice were glycerol intolerant, with a 1.4-fold increase in glycemic excursion (*P* < 0.05) (Figure 4D). At this time point, there was no difference between KO mice and controls with respect to glycemic excursion during a glucose tolerance test (Figure 4E), suggesting that young HGFAC-KO animals may have dysregulated hepatic glucose production. However, after 13 weeks of HF/HS diet, HGFAC-KO mice developed glucose intolerance with a 1.6-fold increase in incremental area under the curve (iAUC, *P* < 0.005), as well as insulin resistance with a 30% decrease in incremental area above the curve (iAAC, *P* < 0.05), as measured by IP glucose and insulin tolerance tests, respectively (Figure 4, F and G). HGF has been proposed to regulate pancreatic β cell development and insulin secretory capacity (45). To test insulin secretory capacity in HGFAC-KO mice, we performed an oral mixed meal tolerance test, which triggers more robust and sustained insulin secretion than IP glucose administration. Basal insulin and glucose levels were not different between HGFAC-KO mice and controls (Figure 4H). At 10 minutes, insulin levels were 1.6-fold higher in HGFAC-KO mice compared with controls (3.37 ± 0.48 ng/mL HGFAC KO vs. 2.1 ± 0.4 ng/mL controls, *P* < 0.05), with only a modest increase in glycemia at this time point. These data indicate that HGFAC-KO mice subjected to HF/HS diet develop early dysregulated hepatic glucose production followed by systemic insulin resistance with intact insulin secretory capacity.

We also examined whether HF/HS diet might exacerbate the increase in circulating triglyceride levels observed in chow-fed HGFAC-KO mice. In contrast with the data in chow diet, we did not observe consistent differences in circulating triglycerides after 7–8 weeks of HF/HS diet in most study cohorts. We observed higher circulating triglycerides and a trend toward increased cholesterol on HF/HS diet only in one additional cohort (Supplemental Figure 3, A and B). Similarly, triglyceride levels were not different between HGFAC KO and control after IP administration of poloxamer 407, which inhibits lipoprotein lipase and peripheral triglyceride clearance (Supplemental Figure 3C), indicating that VLDL production is similar between genotypes in this dietary context (46).

### HGFAC KO downregulates hepatic PPARγ expression.

**Figure 5:**
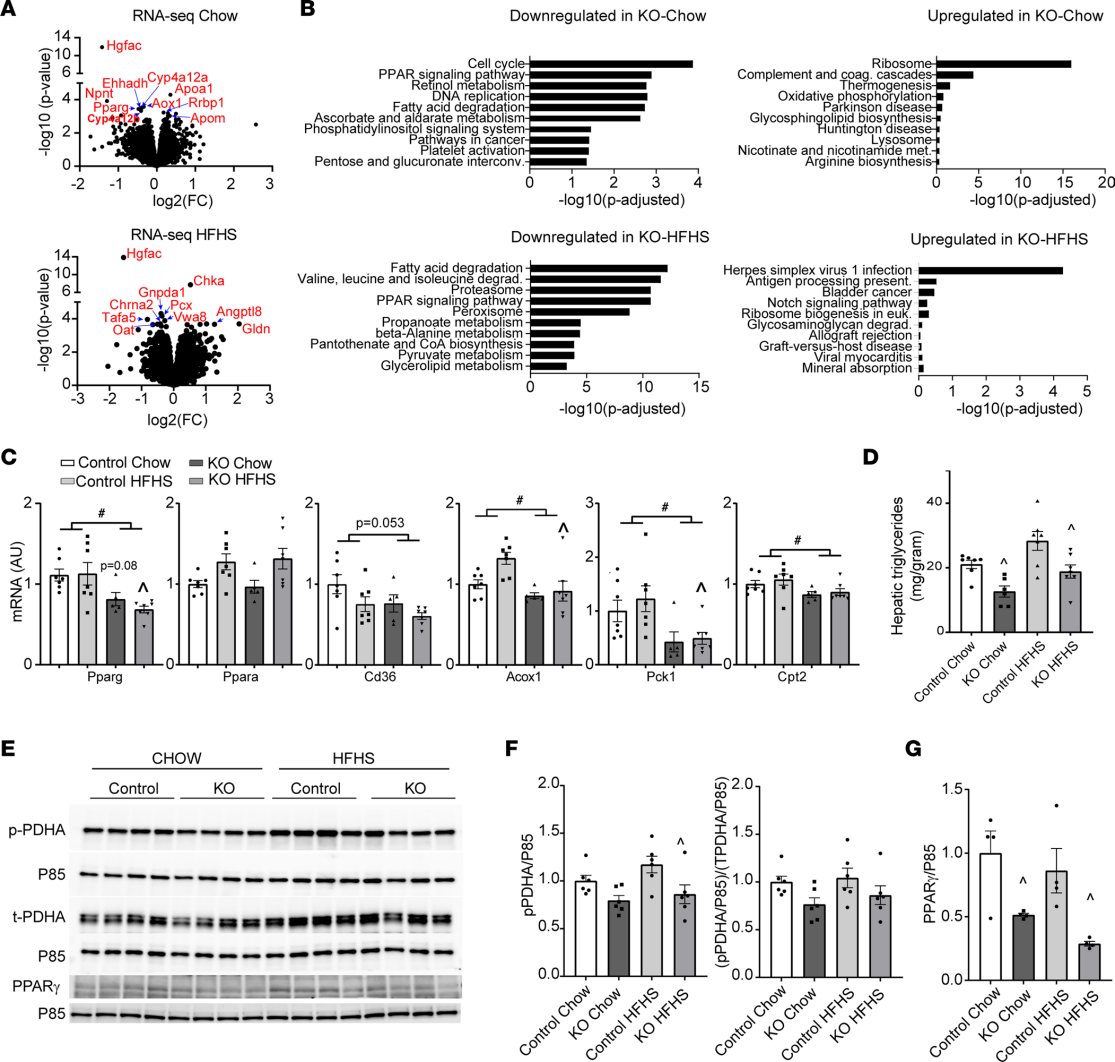
Hepatic PPARγ is downregulated in HGFAC-KO mice. (**A**) Volcano plot depicting differentially expressed genes from livers of chow- and HF/HS-fed HGFAC-KO mice versus controls. Named genes in red represent top 10 most differentially expressed genes ranked by *P* value. (**B**) Pathway analysis including the top 10 most downregulated and upregulated gene sets, respectively, in chow and HF/HS-fed HGFAC-KO livers compared with controls. (**C**) Hepatic mRNA levels of Pparg, Ppara, Cd36, Acox1, Pck1, and Cpt2 after 4 weeks of chow or HF/HS diet (*n* = 5–7/group). (**D**) Hepatic triglyceride levels in control and HGFAC-KO mice on chow and HF/HS diet after overnight fasting followed by 4-hour ad libitum refeeding (*n* = 6–7/group). (**E**) Immunoblot analysis and quantification of hepatic phospho-S293 PDHA, total PDHA and PPARγ, and P85 loading control in chow- or HF/HS-fed HGFAC KO and controls with quantification of phospho-S293 PDHA normalized to (**F**) P85 or to total PDHA (*n* = 6/group) and (**G**) PPARG normalized to P85 (*n* = 4/group). Data represent means ± SEM. Statistics were assessed by 2-way ANOVA with Holm-Šídák multiple comparisons between individual groups, ^#^*P* < 0.05, for genotype main effects, ^*P* < 0.05 for comparison across genotypes within diets.

To define mechanisms that might contribute to altered triglyceride and carbohydrate metabolism in HGFAC-KO mice, we performed RNA-Seq analysis on liver from chow- and HF/HS-fed HGFAC-KO mice and littermate controls after 4 weeks on the diet. *Hgfac* was the most significantly downregulated mRNA on both diets, validating successful KO (Figure 5A). By pathway enrichment analysis (Figure 5B), genes involved in cell cycling were the most downregulated set in chow-fed HGFAC-KO mice. This is consistent with HGF’s known effects to stimulate hepatocyte proliferation (47). Pathway analysis also suggested changes in lipid metabolism with reduced “PPAR signaling pathway” and “Fatty acid degradation” in KO mice on both diets. Upregulation of genes involved in ribosomal function were observed in the HGFAC-KO mice, potentially consistent with reduced cell cycling and enhanced differentiated function as a result of reduced HGF signaling. Gene sets associated with complement and coagulation pathways were also upregulated in HGFAC-KO mice. Upregulation of complement and coagulation pathways is notable as putative loss-of-function variants in the *HGFAC* locus also associate with increased circulating fibrinogen levels (48).

Consistent with the pathway analysis, *Pparg* was in the top 10 most differentially expressed genes comparing chow-fed HGFAC-KO mice and controls (Supplemental Table 3). To validate this, we quantified hepatic mRNA gene expression by qPCR, which revealed that *Pparg* but not *Ppara* was downregulated in livers of chow- and HF/HS-fed HGFAC-KO mice compared with controls (Figure 5C). Furthermore, PPARγ target genes were downregulated. These results were replicated in a second cohort (Supplemental Figure 4A). Surprisingly, we found that downregulation of *Pparg* and its targets was liver specific, as subcutaneous adipose tissue (inguinal) expression of *Pparg* and *Cd36* was similar between HGFAC-KO and control mice (Supplemental Figure 4B). Hepatic PPARγ is reported to enhance liver fat accretion yet preserve hepatic and systemic insulin sensitivity (49, 50). HF/HS feeding increased the levels of hepatic triglycerides by 49% and 34% in HGFAC-KO mice and controls, respectively (Figure 5D). However, hepatic triglyceride levels were reduced by 40% and 32% in HGFAC-KO mice compared with controls on chow and HF/HS diets, respectively. Recently, Shannon et al. reported that pioglitazone, a PPARγ agonist, inhibits the activity of catalytic subunit E1α of hepatic pyruvate dehydrogenase (PDHA) and diminishes hepatic glucose output but increases the level of hepatic triglycerides (51). Consistent with this mechanism, we observed reduced inhibitory S293-PDHA phosphorylation in HGFAC-KO animals on chow and HF/HS diets, indicative of increased PDHA activity. Moreover, hepatic PPARγ protein levels were reduced in HGFAC-KO mice in both chow- and HF/HS-fed conditions, indicating diminished PPARγ activity (Figure 5, E–G). Phenotypes in HGFAC-KO mice were consistent with liver-specific deletion of PPARγ, which results in reduced hepatic steatosis and impaired hepatic glucose homeostasis eventually leading to the development of peripheral insulin resistance (49, 50). This may be in part mediated by the effects of PPARγ on hepatic PDHA activity.

### HGFAC overexpression enhances glucose homeostasis.

**Figure 6:**
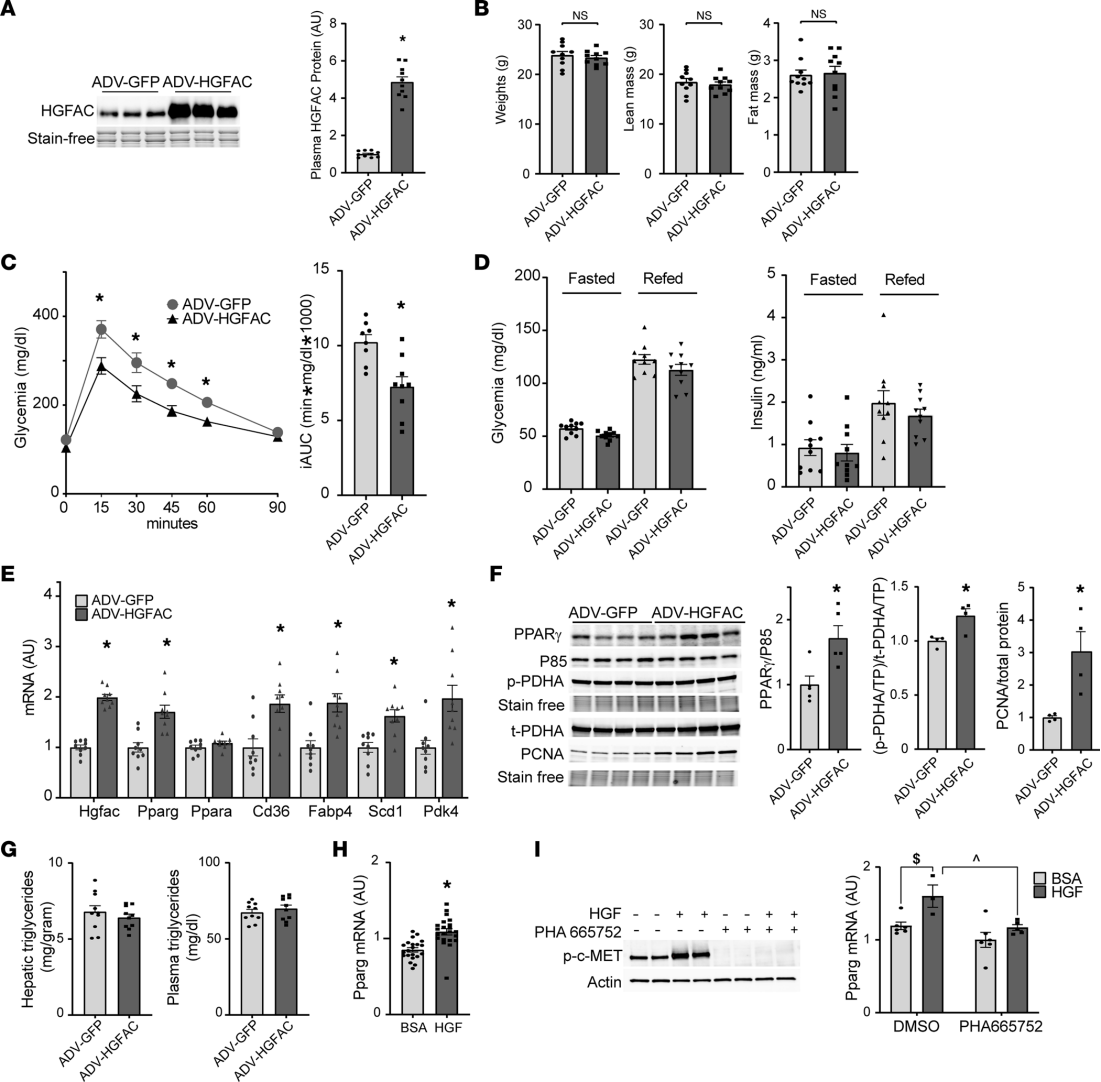
HGFAC overexpression enhances glucose homeostasis. (**A**) Immunoblot and quantification by densitometry of plasma HGFAC collected 3 days after 8-week-old male mice were transduced with adenovirus expressing GFP (ADV-GFP) or HGFAC (ADV-HGFAC). (**B**) Weights and lean and fat mass of ADV-GFP and ADV-HGFAC mice after 9 days of transduction (*n* = 10/group). (**C**) IP glucose tolerance test and corresponding iAUC performed 5 days after viral transduction (*n* = 8–9/group). (**D**) Overnight-fasted and 3-hour refed glycemia and peripheral insulin levels of GFP- and HGFAC-transduced mice (*n* = 10). (**E**) Hepatic mRNA levels of *Hgfac*, *Pparg* and -*a*, and PPARγ targets measured by qPCR 14 days after viral transduction. (**F**) Hepatic PPARγ, phospho-S293 PDHA, total PDHA, and PCNA immunoblots of liver from ADV-HGFAC– and ADV-GFP–transduced mice and quantification of PPARγ normalized to P85, phosphorylated PDHA normalized to total PDHA, and PCNA normalized to the total protein content (*n* = 4–5/group). (**G**) Hepatic and circulating triglyceride levels 14 days after viral transduction in ad libitum–fed mice. (**H**) *Pparg* mRNA levels in AML12 cells after overnight treatment with 50 ng/mL HGF or BSA. (**I**) c-MET phosphorylation by HGF in AML12 cells is inhibited by the c-MET inhibitor PHA-665752 (2.5 μM) preventing induction of *Pparg* mRNA. Data represent means ± SEM. Statistics assessed by 2-tailed unpaired *t* test, *P < 0.05; or by 2-way ANOVA with Holm-Šídák multiple comparisons between individual groups, ^*P* < 0.05 for comparison of effects of inhibitor within HGF treatment condition, ^$^*P* < 0.05 for effect of HGF within inhibitor or control treatment.

As HGFAC deficiency decreased expression of hepatic *Pparg* and its targets, we examined whether HGFAC overexpression has reciprocal molecular and metabolic effects. Adenoviral (ADV) mediated overexpression of HGFAC resulted in a robust increase of circulating HGFAC over 2 weeks compared with ADV-GFP controls (Figure 6A). HGFAC overexpression had no effect on body weight or body composition (Figure 6B) but was associated with markedly improved glucose tolerance, with a 30% reduction in incremental AUC (*P* < 0.005) (Figure 6C) and a 50% reduction in glycemic excursion during a glycerol tolerance test performed in a second cohort (*P* < 0.0005) (Supplemental Figure 5A). Additionally, glucose levels were modestly but significantly lower in ADV-HGFAC mice in the ad libitum–fed condition (*P* < 0.05, 2-tailed *t* test), as well as the overnight-fasted and 3-hour refed conditions (*P* < 0.05, 2-way ANOVA, main effect). However, peripheral insulin levels in fasted and refed ADV-HGFAC mice were not different from the levels of ADV-GFP mice (Figure 6D and Supplemental Figure 5B). The combination of reduced glycemia without changes in insulin following HGFAC overexpression are suggestive of increased insulin sensitivity. Analysis of hepatic gene expression revealed that HGFAC overexpression induced expression of *Pparg* but not *Ppara*, as well as PPARγ target genes such as *Cd36* and *Fabp4* as well as *Pdk4*, which may participate in regulation of PDHA phosphorylation (Figure 6E). Furthermore, complementary to HGFAC-KO mice, HGFAC overexpression increased hepatic PPARγ protein levels and phosphorylation of PDHA (S293), as well as proliferating cell nuclear antigen (PCNA) levels, indicating increased proliferation (Figure 6F). Whereas short-term overexpression of HGFAC was sufficient to produce glycemic and gene expression phenotypes reciprocal to HGFAC KO, we did not observe changes in hepatic or circulating triglyceride levels in this time frame (Figure 6G). Thus, HGFAC overexpression can induce changes in hepatic PPARγ expression and glucose homeostasis independently of its effects on hepatic lipids.

To assess whether HGFAC’s effect to induce *Pparg* expression is likely mediated through its ability to activate HGF and c-MET signaling, we treated murine AML12 hepatocyte-like cells with recombinant, active HGF. HGF treatment increased c-MET phosphorylation and increased *Pparg* mRNA expression by 30% (Figure 6H). These effects were inhibited by pretreatment with PHA-665752, a c-MET inhibitor (Figure 6I) (52). These results support a model whereby overnutrition enhances ChREBP-dependent upregulation of HGFAC, which activates an HGF/PPARγ signaling axis to preserve systemic glucose homeostasis.

## Discussion

ChREBP is a key transcription factor that is activated in major metabolic tissues by cellular carbohydrate metabolites and mediates genomic and physiological responses to overnutrition. The mechanisms by which carbohydrates activate ChREBP remain controversial (see ref. 1). Putative mechanisms include carbohydrate-mediated translocation of ChREBP protein from the cytosol to the nucleus, alterations in ChREBP posttranslational modifications, and/or allosteric effects of specific carbohydrate metabolites on ChREBP to enhance transactivation. We previously demonstrated that fructose gavage acutely and robustly activates ChREBP-dependent gene expression in mouse liver (4). Here, we performed ChIP-Seq for ChREBP following fructose gavage after a 5-hour fast to map ChREBP binding in mouse liver chromatin. We identified about 4,000 ChREBP binding sites in livers from 2 mouse strains that are similar to previous efforts (10). To our surprise, while fructose acutely activates ChREBP-dependent gene transcription, chromatin-bound ChREBP was readily detectable in fasted animals, and no marked increase in binding was observed following fructose gavage. These results suggest that carbohydrate-stimulated nuclear translocation and accumulation of nuclear ChREBP are not essential for the ability of carbohydrates to enhance ChREBP’s transcriptional activity. These results favor models suggesting that either carbohydrate-mediated posttranslational modification or allosteric activation are the key mechanisms to stimulate ChREBP’s transcriptional activity.

Variants in the human ChREBP locus associate with pleiotropic biological traits with a particularly strong association with hypertriglyceridemia. The transcriptional targets that mediate ChREBP’s pleiotropic biological effects remain incompletely defined. By mapping ChREBP genomic binding sites in mouse liver and integrating them with human genetics data, we identified candidate contributors to ChREBP-mediated regulation of circulating lipids. While genes and loci in proximity to ChREBP binding sites were enriched for variants that associated with hypertriglyceridemia, of the thousands of hepatic ChREBP binding sites, only about 2% of such sites contributed to the enrichment. We anticipate that relatively small subsets of distinct ChREBP gene targets may contribute to its regulation of other metabolic traits.

**Figure 7:**
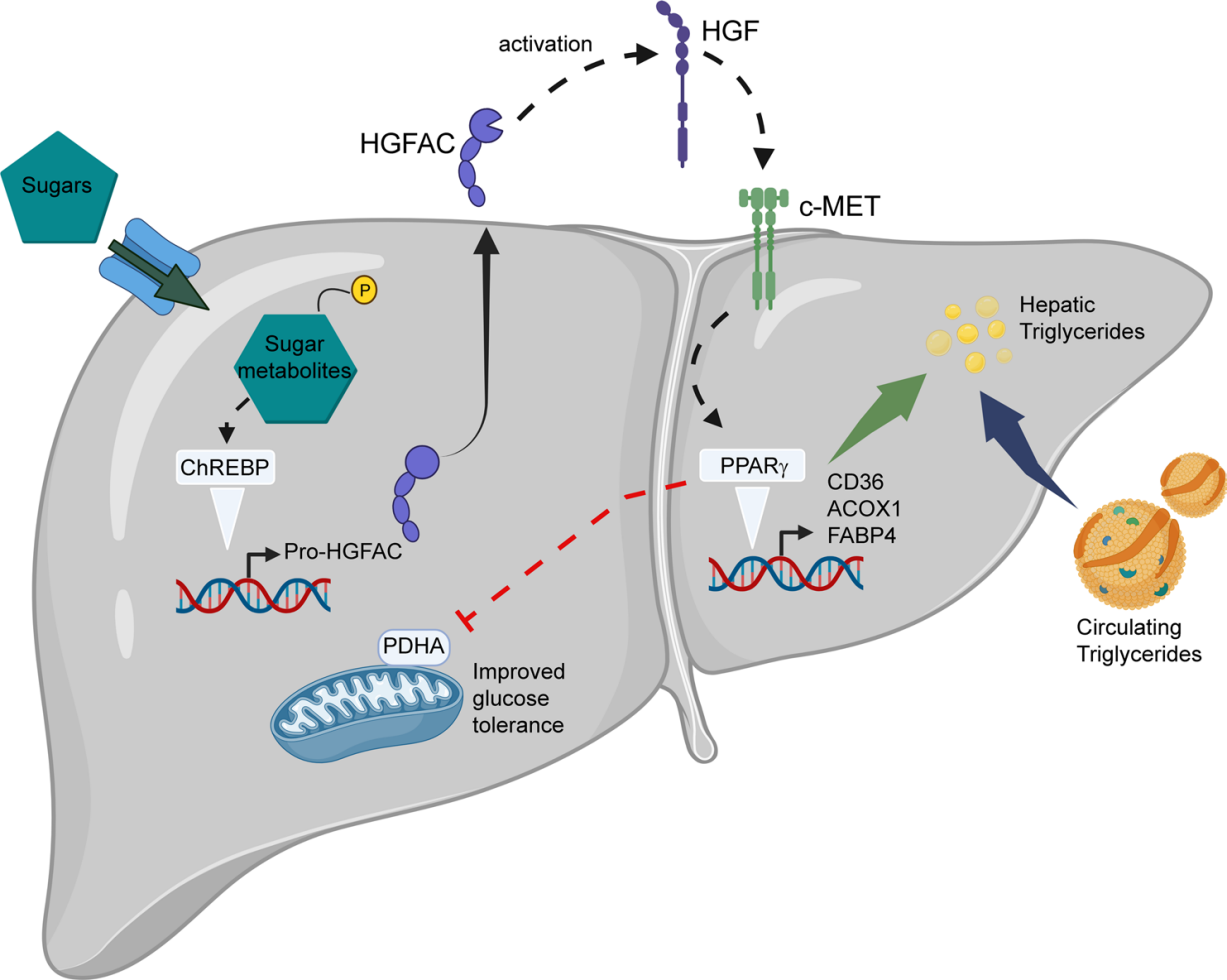
ChREBP-mediated activation of an HGFAC/HGF/PPARγ signaling axis mediates an adaptive response to preserve glucose tolerance in the setting of diets high in sugar. Glucose and fructose from high-sugar diets enhance production of sugar metabolites (hexose-phosphates) in the liver that activate hepatic ChREBP and lead to increased *Hgfac* transcription and translation. HGFAC is secreted into the circulation, where, once activated, it can act in a paracrine or endocrine fashion to proteolytically cleave and activate HGF. HGF binds and activates the c-MET tyrosine kinase receptor on hepatocytes and other cell types. In liver, this leads to upregulation of PPARγ expression that in turn activates transcriptional programs to promote hepatic triglyceride storage and to decrease circulating triglycerides. Additionally, hepatic PPARγ activity decreases activation of the pyruvate dehydrogenase complex, and this contributes to enhance systemic glucose tolerance.

A small minority of candidates were annotated as circulating factors or “hepatokines” that might regulate metabolism systemically. We elected to focus attention on HGFAC as a putative ChREBP-regulated hepatokine and demonstrated that circulating HGFAC is indeed nutritionally regulated in a ChREBP-dependent manner. Moreover, we showed that it participates in an adaptive metabolic response to obesogenic diets in part through its effects to stimulate hepatic *Pparg* expression and transcriptional activity (Figure 7).

To test the role of HGFAC in metabolism, we generated global HGFAC-KO mice. The ability of serum from HGFAC-KO mice to activate HGF and facilitate c-MET signaling was impaired. The attenuation, but not full abrogation, of this activity is consistent with known redundancy in enzymes capable of HGF activation (43, 53). Alternative proteases including kallikreins, urokinases, matriptase, and HPN may compensate for loss of HGFAC activity (53–56).

While *HGFAC* is highly expressed in the liver, it is also expressed at orders of magnitude lower levels in other tissues, including the testes, the intestines, and possibly the pancreatic islets (23, 24, 57). While we cannot rule out the contribution of extrahepatic HGFAC on the observed phenotypes, the majority of HGFAC found in circulation is likely originating from the liver. Indeed, liver-specific ChREBP-KO mice have lower circulating levels of HGFAC, which failed to increase on HFrD. Additionally, ADV overexpression of hepatic HGFAC increased circulating HGFAC levels and produced systemic metabolic effects, further indicating that HGFAC is a ChREBP-regulated hepatokine.

We observed that HGFAC KO reduced and increased hepatic and circulating triglycerides, respectively. This was associated with impaired hepatic and systemic glucose tolerance. While higher circulating triglycerides were present in chow-fed cohorts, this phenotype was not consistently observed with HF/HS diet challenges. One potential explanation is that HF/HS diet through increased fat delivery and storage can overwhelm the subtle effects of HGFAC and hepatic PPARγ on circulating triglycerides in mice. Additional experiments assessing triglyceride secretion and/or clearance will be required to fully explain these observations. ADV-HGFAC overexpression produced a reciprocal phenotype with respect to glucose homeostasis but did not alter liver or circulating lipids in the short time frame of this experiment. Our results contrast with the reported effects of acute treatment with recombinant, active HGF in rodents to reduce steatosis and with inconsistent effects on circulating triglycerides (58, 59). Additionally, marked and sustained transgenic overexpression of HGF under a metallothionein promoter reduced steatosis, in contrast with our observations (60). The differences observed in these publications and our experiments may be due to differences in gain- versus loss-of-function experiments, differential effects in acute versus chronic paradigms, and the degree of changes in HGF activity and signaling.

The specific mechanism by which pro-HGF is activated, either by HGFAC or by other proteases, also appears to have a marked impact on where HGF signaling may be enhanced and on the resultant systemic metabolic effects. As an example, HPN is a membrane-bound protease expressed in multiple tissues that is also capable of HGF activation. HPN KO, which also reduces HGF/c-MET signaling, produces a vastly different metabolic phenotype compared with HGFAC-KO mice. Global HPN-KO mice are resistant to diet-induced obesity, and this lean phenotype is associated with enhanced glucose and lipid homeostasis (61). Profound changes in energy homeostasis in HPN-KO mice and its lean phenotype appear to be due to extensive expansion of brown fat and increased thermogenesis, features that we did not observe in HGFAC-KO mice.

Proteases such as HGFAC and HPN are promiscuous and may activate other peptide hormones, which may also contribute to their differing biological effects. For instance, HGFAC can also cleave and activate pro–macrophage stimulating protein (pro-MSP, also known as MST1) which then activates the RON receptor tyrosine kinase (also known as MST1R) (62, 63). Although we determined that HGF activation and c-MET signaling were impaired in experiments conducted with serum from HGFAC-KO mice, it remains possible that some of the HGFAC-mediated changes that we observed were an effect of decreased signaling through MSP-RON cascade or other, unknown HGFAC proteolytic targets. Nevertheless, concordant associations in human HGFAC and c-MET variants with phenotype in HGFAC-KO mice indicate that some of the key biological effects observed in HGFAC-KO mice are likely mediated through reduced HGF/c-MET signaling.

Our results show that the ChREBP/HGFAC axis regulates hepatic PPARγ signaling in mice. We further validated this observation by showing that HGF treatment could increase *Pparg* expression in hepatocyte-like AML12 cells and this could be blocked by a c-MET inhibitor. While the metabolic role of PPARγ is most well recognized with respect to adipogenesis, hepatic PPARγ also appears important in regulating systemic metabolism (64–66). Liver-specific deletion of *Pparg* reduces steatosis but leads to hypertriglyceridemia and glucose intolerance associated with muscle and adipose insulin resistance (49). While the beneficial effects of hepatic PPARγ have been attributed to its effects on reducing circulating lipids, recent work demonstrated that the PPARγ agonist pioglitazone enhances hepatic insulin sensitivity independently of its effects on hepatic lipids and is instead dependent on PPARγ’s ability to inhibit hepatic pyruvate dehydrogenase (PDH) activity (51). Data from HGFAC-KO mice are consistent with this hypothesis in that decreased PPARγ activity is accompanied by a reduction in inhibitory phosphorylation of the PDH catalytic subunit on Ser293. ADV overexpression of HGFAC led to marked improvement in glucose tolerance with increased hepatic PPARγ expression and increased phosphorylation of PDH consistent with this model. While HGFAC overexpression increased hepatic PDH kinase 4 mRNA levels, we did not detect a reciprocal decrease in PDH kinase 4 expression in HGFAC-KO mice, suggesting that other kinases and/or phosphatases may mediate HGFAC-induced changes in PDHA phosphorylation. Interestingly, recent work by Huang et al. suggests that the HGF receptor, c-MET, itself can phosphorylate and inactivate PDHA by direct interaction (67). While we have not tested the putative direct interaction between c-MET and PDHA in our models, this work supports our observations, indicating that increased HGF signaling via HGFAC activity leads to inhibition of the PDH complex. Together, our results indicate that HGF and PPARγ may mediate some of HGFAC’s effects on glucose homeostasis through regulation of hepatic PDH phosphorylation.

Putative loss-of-function variants in human HGFAC associate with increased circulating triglycerides, albumin, and platelets, and these phenotypes are recapitulated in HGFAC-KO mice (34). This concordance supports the hypothesis that putative HGFAC loss-of-function variants likely impair its catalytic activity. Moreover, these results suggest that this molecular physiology is conserved from rodents to humans. Interestingly, the rs1801282 (Pro12Ala) PPARG variant associated with increased *PPARG* expression and reduced risk for diabetes and circulating triglycerides also associates with reduced albumin levels (68). These effects on albumin are directionally concordant with the changes in albumin that occur in HGFAC-KO mice and the reduction in hepatic *Pparg*. Again, this suggests that an HGF/PPARγ signaling axis is conserved in humans and that some of the beneficial effects of PPARγ on systemic metabolism could be mediated through effects in the liver in addition to adipose tissue.

Our results suggest an integrated physiology whereby carbohydrate sensing via ChREBP impacts systemic growth factor signaling (HGFAC/HGF/c-MET) that may mediate both adaptive and maladaptive responses through paracrine and endocrine effects. In the context of obesogenic diets, this signaling axis enhances hepatic *PPARG* expression, which may mediate a compensatory response to preserve systemic glucose homeostasis. HGF, the principal target for HGFAC, has previously been implicated in other aspects of glucose homeostasis. For example, HGF may enhance pancreatic β cell proliferation (45, 69–71). Increased ChREBP-mediated HGFAC secretion might be a potential mechanism to increase β cell mass in the setting of increased dietary carbohydrate burden. Additionally, within the liver, HGF has been reported to enhance insulin signaling and hepatic glucose clearance via physical interactions between its receptor, c-MET, and the insulin receptor (72). HGF also is secreted by adipocytes and can promote angiogenesis in adipose tissue, and adipose angiogenesis is an integral feature of adipose tissue expansion (73–75). Therefore, elevated ChREBP/HGFAC/HGF may promote healthy expansion of adipose tissue for efficient storage of fuel during overnutrition. These observations may support a role for ChREBP-mediated upregulation of HGFAC and HGF signaling as an adaptive response to increased nutritional burden and will require further investigation. ChREBP itself has been shown to regulate mouse hepatocyte and murine and human β cell proliferation (76–78). The ChREBP/HGFAC axis may provide an important mitogenic signal through HGF when ChREBP senses abundant carbohydrates indicative of ample building blocks supporting proliferation. Our data support this hypothesis, as HGFAC-KO animals have decreased expression of hepatic cell cycle genes, and ADV overexpression of HGFAC leads to marked upregulation of PCNA in the liver, a marker of proliferation.

Putative loss-of-function variants in HGFAC associate with increased circulating HGF in humans and associate with increased cardiovascular risk factors (32, 34). Increased circulating HGF itself is increasingly recognized as a cardiometabolic risk factor that may be independent of other canonical cardiovascular risk factors (28, 30, 32, 79, 80). Further investigation into the relationship between ChREBP, HGFAC, and HGF signaling may define new mechanisms contributing to the pathogenesis of cardiometabolic disease in humans.

## Methods

### Reagents.

We used glucose (catalog 8769), glycerol (catalog G2025-1L), poloxamer 407 (catalog 16758-250G), and dextran sulfate (catalog D8906-5G) from MilliporeSigma; Ensure Original Nutritional Shake from retail pharmacy; PHA-665752 (catalog 14703) from Cayman Chemical; mouse recombinant active HGF protein (catalog 2207-HG) from R&D Systems, Bio-Techne; mouse Ultra-Sensitive Insulin ELISA from Crystal Chem (catalog 90080); Triglyceride LiquiColor test (catalog 2200225) from StanBio Laboratories; total cholesterol (catalog 999-02601) and NEFA-HR(2) (Wako); and thrombin (catalog T4648-1KU) from MilliporeSigma.

### Animals and diets.

Floxed ChREBP mice were generated at UT Southwestern Medical Center as previously described (12). Albumin-Cre mice (stock 003574) were purchased from The Jackson Laboratory. ChREBP LKO experiments were performed on a mixed C3H/HeJ and C57BL/6J background as previously described (3). HGFAC-KO mice on a C57BL/6J background were generated at the Duke Transgenic and Knockout Mouse Core, by introducing an 857 bp spanning mid exon 1 and exon 2 by CRISPR/Cas9. ADV overexpression of HGFAC was performed in wild-type C57BL/6J male mice purchased from The Jackson Laboratory. Mice were fed a chow diet (LabDiet 5008 or 5053), 60% fructose diet (TD.89247 Harlan Teklad), or 45% fat/18% sucrose diet (D12451i, Research Diets) ad libitum for indicated times. Experimental mice were housed at 21°C–22°C on a 12-hour light/12-hour dark cycle in ventilated cages with 30 air exchanges per hour. All experiments were conducted with male mice, except where stated otherwise. Genotyping primers can be found in Supplemental Table 4. Rat experiments were performed in double-housed, 8-week-old, male Wistar rats (Charles River Laboratories) maintained on a standard chow diet (TD.7001, Harlan Teklad). Rats were fasted overnight and then fed either standard chow or 60% fructose diet (TD.89247, Harlan Teklad) ad libitum. Rats were sacrificed 4 hours later, and livers were snap-frozen for further analysis.

### Cell lines.

AML12 (CRL-2254) and HepG2 (HB-8065) cells were obtained from ATCC. AML12 cells were cultured in DMEM/F12 + 10% FBS supplemented with 1× Insulin-Transferrin-Selenium (100×, Thermo Fisher Scientific, 41400045) and 40 ng/mL dexamethasone (MilliporeSigma, D4902). HepG2 cells were cultured in DMEM + 10% FBS (Thermo Fisher Scientific, 16000044).

### ChIP-Seq and analysis.

Wild-type, male, 8-week-old C3H/HeJ and C57BL/6J mice were fasted for 5 hours and gavaged with fructose (4 g/g BW) versus water control (*n* = 6/group). Mice were euthanized 90 minutes after gavage, and tissues were harvested and snap-frozen in liquid nitrogen for further analysis. Chromatin was prepared using truChIP Chromatin Shearing Tissue Kit (Covaris). A total of 25–30 mg of frozen liver tissue was quickly minced with razor blades in PBS at room temperature. Tissue was crosslinked with 0.5 M disuccinimidyl glutarate in PBS for 45 minutes at room temperature, followed by fixation with 1% formaldehyde in Fixing Buffer A (Covaris) for 5 minutes at room temperature. Crosslinking was stopped by Quenching Buffer E (Covaris). After washing, nuclei were isolated by Dounce homogenization followed by centrifugation at 1,700*g* for 5 minutes at 4°C. The nuclear pellet was resuspended in cold 0.25% SDS Shearing Buffer (Covaris). Chromatin was sheared in 1 mL AFA milliTUBEs (Covaris) using Covaris S220X focused ultrasonicator with the following parameters: peak incident power 140 W, duty factor 5%, and cycles per burst 200 for 12 minutes. The sheared chromatin was centrifuged 15,800*g* for 15 minutes at 4°C to pellet the debris, and a 10 μL aliquot was de-crosslinked and used for quantification with Qubit (Thermo Fisher Scientific). Sheared chromatin (1.5–3 μg) was diluted in ChIP dilution buffer (16.7 mM Tris [pH 8], 1.2 mM EDTA, 25 mM NaCl, 1.1% Triton X-100, 0.01% SDS), and 1 μg of ChREBP antibody (Novus Biologicals, Bio-Techne, catalog NB400-135) or control rabbit IgG (sc-2027, Santa Cruz Biotechnology) was added, followed by overnight incubation at 4°C. Reactions were then incubated for 1 hour at 4°C with protein A/G dynabeads (Invitrogen, Thermo Fisher Scientific) preblocked in PBS/0.5% and BSA/0.5% Tween. Beads were then washed in low-salt wash buffer (20 mM Tris [pH 8], 1 mM EDTA, 140 mM NaCl, 1% Triton X-100, 0.1% sodium deoxycholate, 0.1% SDS), high-salt wash buffer (20 mM Tris [pH 8], 1 mM EDTA, 500 mM NaCl, 1% Triton X-100, 0.1% sodium deoxycholate, 0.1% SDS), LiCl wash buffer (10 mM Tris [pH 8], 1 mM EDTA, 0.5% NP-40, 0.5% sodium deoxycholate, 250 mM LiCl), and TE buffer (10 mM Tris [pH 8], 1 mM EDTA) and eluted and reverse crosslinked in elution buffer (10 mM Tris [pH 8], 5 mM EDTA, 0.1% SDS, 300 mM NaCl, 0.8 mg/mL proteinase K, 10 μg/mL RNase A) by incubating at 65°C for 10 hours. DNA was extracted using AMPure XP beads following the manufacturer’s manual and quantified by Qubit (Thermo Fisher Scientific). Immunoprecipitated chromatin was pooled by genotype and gavage condition for further analysis.

Library preparation, sequencing, and analysis were performed in the Boston Nutrition Obesity Research Functional Genomics and Bioinformatics Core. The ChIP-Seq reads were demultiplexed using bcl2fastq and aligned to the GRCm38 mouse genome using Bowtie2 (81). PCR duplicates and low-quality reads were removed by Picard. Reads were processed using SAMtools and subjected to peak calling with MACS2. SAMtools was also used to obtain 2 pseudoreplicates per sample (82, 83). Only the peaks present in both pseudoreplicates were included for further downstream analysis. The coverage for peaks was obtained using BEDtools multicov (84). Normalization and differential analysis were performed using edgeR between fructose and water gavage conditions (85). To visualize ChIP-Seq signals, reads were converted to the BigWig file format using BEDtools and bedGraphToBigWig (86). Peaks were tied to genes based on the nearest gene and transcription start site within a radius of 200 kb distance. The gtf file from GENCODE version M24 was filtered to include only processed transcript and protein coding transcript types as well as filtered for well-supported transcripts (using only transcript support levels 1 and 2).

For MAGENTA analysis, genes included in the analysis were further filtered for transcriptional start sites that resided within 20 kb of a ChIP-Seq peak. Human homologs of this set of mouse genes were analyzed using the MAGENTA algorithm in conjunction with joint Metabochip and GWAS triglyceride data from the Global Lipids Genetics Consortium (21, 27). Candidate genes were called secretory proteins based upon their annotation in the UniProt database (87).

### ChIP-PCR.

Male ChREBP-LKO mice on C3H background and littermate controls were fasted overnight and fed HFrD for 3 hours (*n* = 3/group). Mice were euthanized and tissues were harvested and snap-frozen in liquid nitrogen for further analysis. ChIP was performed as above. qPCR was performed as described below.

### Metabolic testing.

Body composition was measured by Bruker Minispec LF 90II. Circulating triglycerides were measured from ad libitum–fed mice at 1 pm in blood collected from the tail vein. For glucose and glycerol tolerance tests, mice were fasted for 5 hours starting at 7 am, and glycerol or glucose (2 g/kg body weight) was injected intraperitoneally. For insulin tolerance tests, mice were fasted overnight, and 1 U insulin/kg (Humulin R, Eli Lilly and Co.) was injected intraperitoneally. Glucose measurements were performed using a handheld glucometer (Bayer Contour). For mixed meal tolerance tests, mice were fasted overnight and gavaged with 10 μL/g of Ensure. Blood was collected from the tail vein at 0- and 10-minute time points for insulin measurement. For VLDL secretion assay, mice were fasted for 3 hours and injected with 1 g/kg poloxamer 407.

### Hepatic triglyceride measurements.

Liver neutral lipids were extracted with a modified Folch method. First, 100 mg of liver tissue was homogenized in 3 mL chloroform/methanol (2:1) and incubated overnight with shaking. Next, 800 μL of 0.9% saline was added, vortexed, and centrifuged (2,000*g* for 10 minutes at room temperature). The chloroform phase was collected and dried overnight. Triglycerides were dissolved in butanol/Triton X-100/methanol (60:27:13 by volume) and measured using colorimetric triglyceride assay (StanBio).

### HGFAC/HGF activation assay.

Blood was collected from 3 control and 3 HGFAC-KO mice and allowed to clot at room temperature for 1 hour, then centrifuged at 7,000*g* for 15 minutes, and serum was collected. Serum was incubated with 10 μg/mL dextran sulfate and 1 U of thrombin for 3 hours at 37°C with 0.05 M Tris, 0.05 M NaCl, and 0.05 M CaCl_2_. HepG2 cells were treated with serum diluted with DMEM (1:10) for 5 minutes and then harvested. Activation of c-MET was assessed by immunoblotting.

### Mouse complete blood count.

Mouse complete blood count was performed with K_2_ EDTA–treated plasma obtained from tail veins via an Element HT5 veterinary hematology analyzer (Duke University Veterinary Diagnostic Laboratory).

### Immunoblotting.

Whole liver tissues were homogenized in lysis buffer containing 20 mM Tris-HCl, 150 mM NaCl, 1 mM Na_2_EDTA, 1 mM EGTA, 1% Triton, phosphatase (Pierce, Thermo Fisher Scientific, A32957), and protease inhibitors (MilliporeSigma, P8340). Protein concentration was measured with the BCA method (Thermo Fisher Scientific, 23225). Approximately 15–40 μg of protein was used for liver immunoblots. For plasma samples, 1 μL of plasma was mixed directly with 15 μL of Laemmli buffer with reducing reagent added (NuPAGE Sample Reducing Agent, NP0004). Lysates were then subjected to immunoblotting with the indicated antibodies: anti-HGFAC (R&D Systems, Bio-Techne, AF1715), anti–β-actin (Cell Signaling Technology, 4970S), anti–phosphorylated c-MET (Cell Signaling Technology 3077), anti–total c-MET (Cell Signaling Technology 3127), anti-PPARγ (Cell Signaling Technology 2435), anti-PDHA1 (phospho-S293) (Abcam, ab92696), anti-PDH (Cell Signaling Technology 3205), anti-p85 (Upstate, 06-496), and anti-PCNA (Cell Signaling Technology, 2586). Quantification of blots was performed with a ChemiDoc XP (Bio-Rad) and Image Lab software v6.0. For loading normalization, whole-lane protein was quantified using Bio-Rad Stain-Free technology.

### qPCR.

TRI reagent (MilliporeSigma, T9424) was used for RNA isolation from mouse liver and cell lines. RNA was reverse-transcribed using a SuperScript VILO kit (Invitrogen, Thermo Fisher Scientific). Gene expression was analyzed with the ABI Prism sequence detection system (SYBR Green; Applied Biosystems, Thermo Fisher Scientific). Gene-specific primers were synthesized by Thermo Fisher Scientific (Supplemental Table 4). Each sample was run in duplicate and normalized to *Tbp* (ChREBP-LKO cohorts), *Ppib* (HGFAC cohorts), or *rplp0* for rat experiments.

### ADV overexpression of HGFAC in mice.

Murine *Hgfac* cDNA (Sino Biological, catalog MG50039-M) was subcloned via Gateway recombination into the pAd/PL-DEST adenoviral vector with CMV promoter (Thermo Fisher Scientific, catalog V49420). The ADV-GFP control vector has been previously described (88). ADV vectors were produced and purified as previously described (88). Anesthetized mice were injected with 5 × 10^10^ ADV particles expressing HGFAC versus GFP control via retro-orbital injection. Expression of HGFAC was assessed by immunoblotting plasma for circulating HGFAC 3 days after ADV transduction.

### RNA-Seq and analysis.

RNA was isolated from mouse liver with TRI reagent (MilliporeSigma, T9424). RNA-Seq was performed at Duke Center for Genomic and Computational Biology. RNA quality was assessed using a Fragment Analyzer (Advanced Analytical Technologies). mRNA capture, fragmentation, and cDNA library construction were conducted using a stranded mRNA-Seq Kit (Kapa Biosystems, KR096, v6.17). 50 bp paired-end sequencing was performed on an Illumina NovaSeq 6000, and at least 35 million reads were obtained per sample. Sequencing data were uploaded to https://usegalaxy.org/ and aligned with HISTAT2 (2.1.0) using mouse genome assembly GRCm38 (mm10). Transcript levels were quantified using FeatureCounts (89). Transcript level count was uploaded to the BioJupies server and analyzed for differential gene expression and Kyoto Encyclopedia of Genes and Genomes pathway enrichment (90–92).

### Human hepatic HGFAC gene expression and analysis.

*HGFAC* mRNA expression values for lean, obese, and obese/diabetic patients were extracted from data deposited in NCBI Gene Expression Omnibus (GEO) (GSE15653) (41). Liver RNA-Seq read counts were obtained from the GTEx project (version 8, 2017-06-05). Genes with average expression value > 20 were log-transformed and transformed to *z* scores. Pearson’s correlations were calculated for each gene with HGFAC. The top 5% of correlated genes were analyzed with enrichR against ARCHS4 transcription factor coexpression database (40, 93). For correlation between *HGFAC* and ChREBP targets, a composite expression vector for validated ChREBP targets (*PKLR*, *ALDOB*, *FASN*, *KHK*, and *SLC2A2*) was computed by averaging the log-transformed *z* score expression values for each of these genes.

### Genomic data.

Genomic data have been deposited in GEO (GSE217983).

### Statistics.

All data are presented as the mean ± SEM. Data sets were analyzed for statistical significance with GraphPad Prism using 2-tailed unpaired *t* tests, and where indicated 2-way ANOVA and with post hoc comparisons performed with Holm-Šídák test or 1-way ANOVA with Holm-Šídák multiple comparisons test between control and individual groups. Statistical significance was set at *P* < 0.05.

### Study approval.

All rodent studies were approved by the Beth Israel Deaconess Medical Center Institutional Animal Care and Use Committee or the Duke University Medical Center Institutional Animal Care and Research Advisory Committee.

## Author contributions

MAH, AS, LD, SAH, and IA designed, performed, and interpreted mouse experiments. PJW and SAH performed rat experiments. MAH, LD, AS, WT, HS, RI, and LTT designed, performed, and interpreted computational analyses. AS and MAH designed, performed, and interpreted in vitro experiments. JMH designed and performed construction of ADV vectors, and MA and PJW prepared purified adenoviruses. PAG, WT, RM, and HHK assisted with performing and interpreting experiments. MAH conceived of, designed, and supervised the experimental plan and interpreted experiments. AS and MAH wrote the manuscript. All authors edited the manuscript.

## Supplementary Material

Supplemental data

Supplemental table 1

Supplemental table 2

Supplemental table 3

Supplemental table 4

## Figures and Tables

**Table 1:**
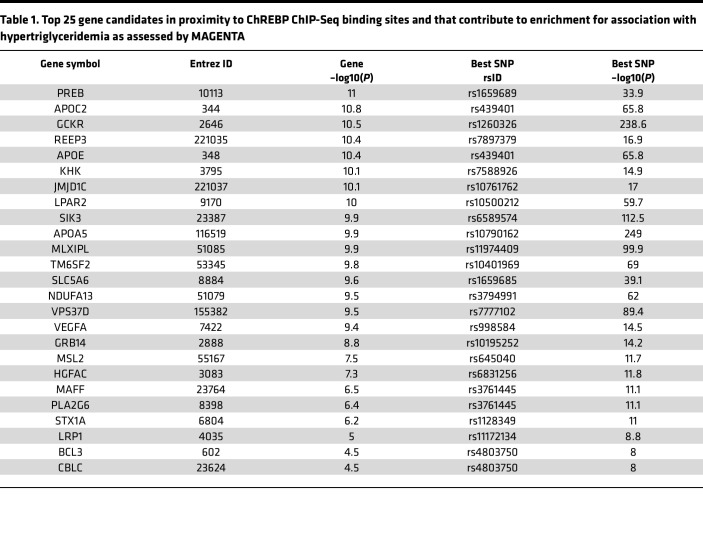
Top 25 gene candidates in proximity to ChREBP ChIP-Seq binding sites and that contribute to enrichment for association with hypertriglyceridemia as assessed by MAGENTA

## Version 1 11/22/2022

In-Press Preview

## Version 2 01/10/2023

Electronic publication
