# Supplementary material for: HGFAC is a ChREBP-regulated hepatokine that enhances glucose and lipid homeostasis
Source: JCI Insight. 2023 Jan 10;8(1):e153740. doi: 10.1172/jci.insight.153740 (PMC9870088; doi:10.1172/jci.insight.153740)
Supplement: Supplemental data [file jciinsight-8-153740-s150.pdf]

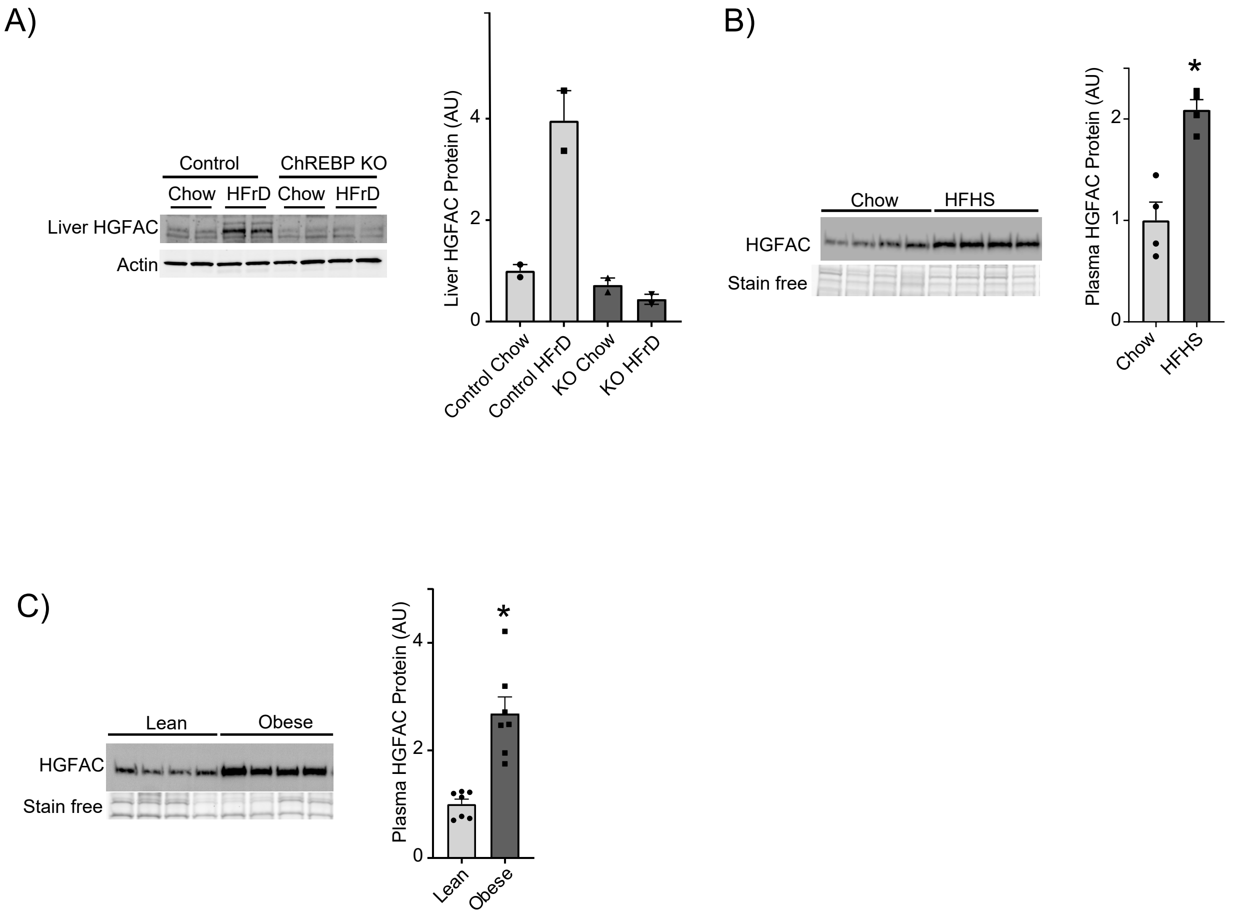

Supplementary figure 1. **A)** Immunoblot analysis and quantification of hepatic HGFAC in control and liver-specific ChREBP KO male mice after 8 weeks on chow versus high fructose diet (HFrD) (n=2/ group). Circulating levels of HGFAC in **(B)** male C57Bl/6J mice after 8 weeks on chow or HFHS diet, (n=4/group), and in **(C)** lean and obese Zucker rats, (n=7/group).(Representative blot shown). Data represent means ± SEM, * $p<0.05$, two-tailed unpaired t-test.

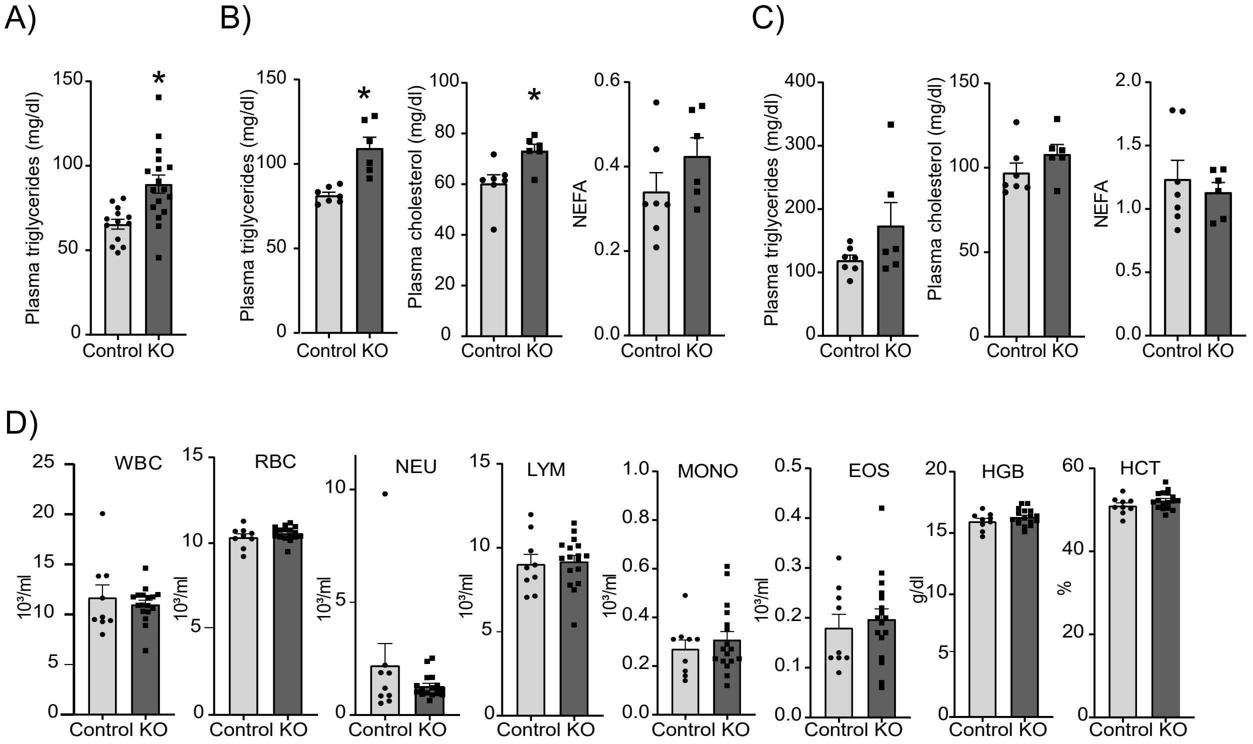

Supplementary Figure 2. A) Circulating levels of triglycerides in adlib-fed female HGFAC KO mice at 11 weeks (n=13-17/group). Circulating levels of triglycerides, cholesterol and NEFA in 8 weeks old control and HGFAC KO male mice on chow diet at ad-lib fed **(B)** and overnight fasted **(C)** state (n=6-7/group). **D)**Numbers of circulating white blood cells (WBC), red blood cells (RBC), neutrophils (NEU), lymphocytes (LYM), monocytes (MONO), and eosinophils (EOS), as well as hemoglobin (HGB) level and hematocrit (HCT) level are similar between Hgfac KO and control mice (n=9-17/group). Data represent means ± SEM,* $p<0.05$; two-tailed unpaired t-test.

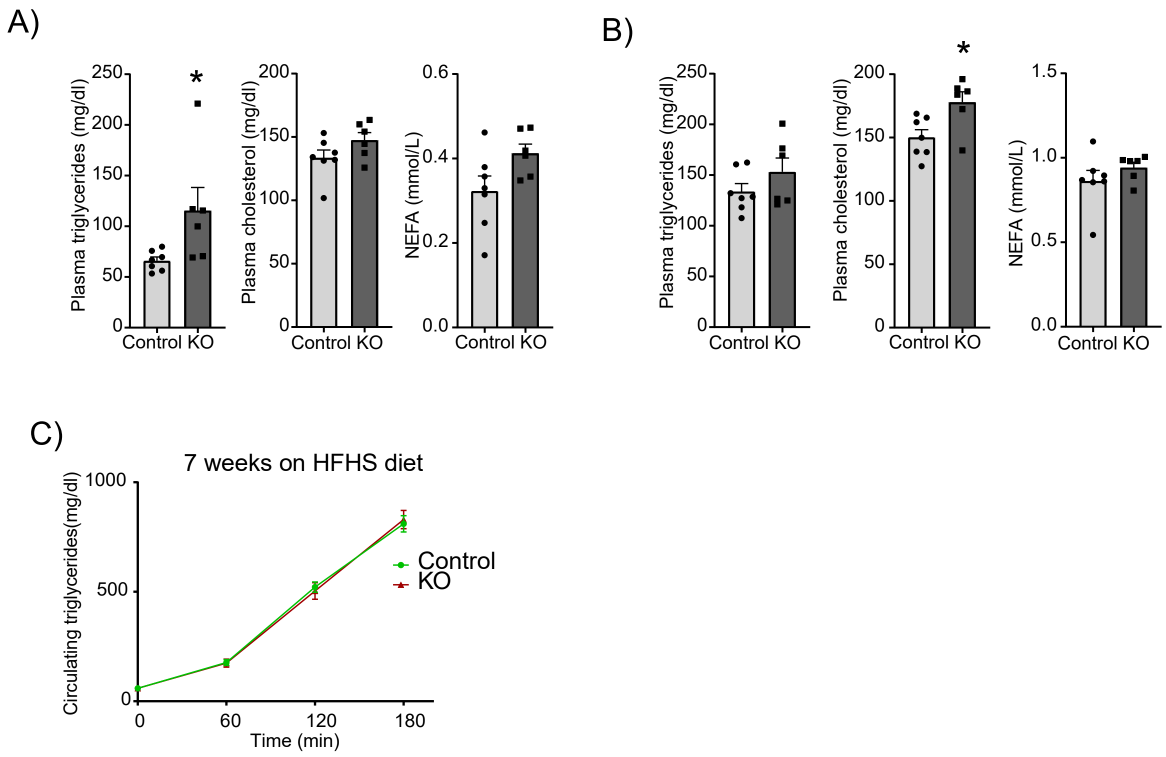

Supplementary Figure 3. Circulating levels of triglycerides, cholesterol and NEFA in control and HGFAC KO male mice after 8 weeks of on HFHS diet at adlib fed **(A)** and overnight fasted **(B)** state (n=6-7/group). **C)**Plasma triglyceride levels in WT and Hgfac KO mice fed HFHS diet for 7 weeks after 3 hours food removal followed by IP injection with 1g/kg poloxamer 407, (n=12/group). Data represent means ± SEM, * p<0.05, two-tailed unpaired t-test.

A)

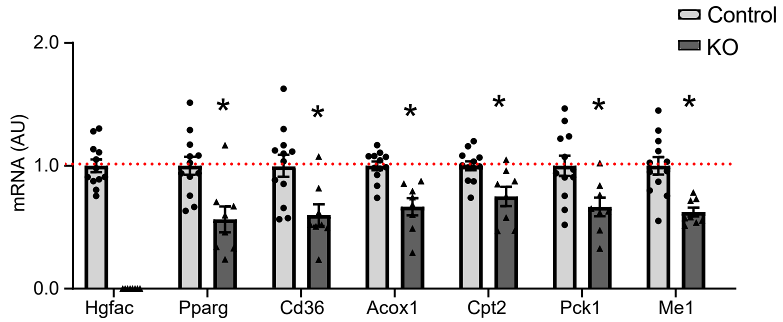

B)

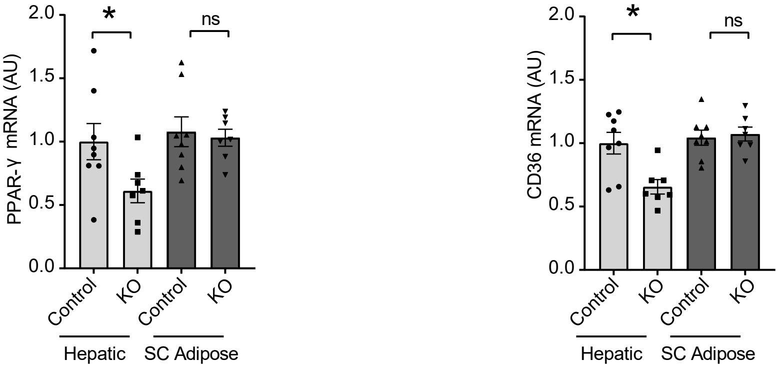

Supplementary Figure 4. A) PPAR-γ and its targets are downregulated in HGFAC KO mice (replication cohort)**.** Hepatic mRNA levels of Hgfac, Pparg, Cd36, Acox1, Cpt2, Pck1 and Me1 in ad libitum chow fed Hgfac KO mice (KO) and controls. (n=8-12/group). **B)**Hepatic, but and SC adipose (Inguinal) tissue expression of Pparg and Cd36 mRNA are downregulated in HGFAC KO animals on Chow diet, (n=7-8/group). Data represent means ± SEM, *p<0.05, two-tailed unpaired t-test.

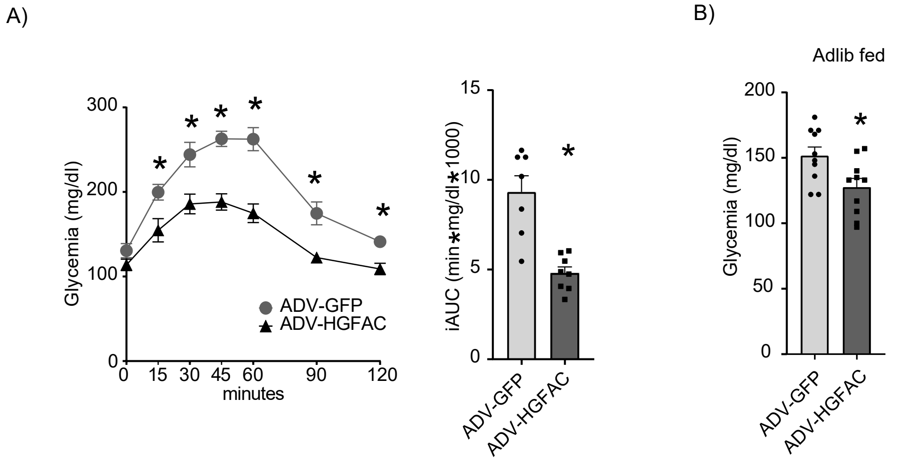

Supplementary Figure 5. A) IP glycerol tolerance test and corresponding iAUC performed two weeks after adenovial transduction, (n=7-8/group). **B)** Adlib-fed peripheral glucose levels in ADV-GFP or ADV-HGFAC transduced mice. n=10 per group, Data represents Mean ± SEM, *p<0.05, two-tailed unpaired t-test.
